# Improving implementation of needle and syringe programmes to expand, scale up, and sustain evidence-based prevention interventions for HIV and hepatitis C in prisons

**DOI:** 10.1016/S2468-2667(24)00275-5

**Published:** 2024-12-16

**Authors:** Nadine Kronfli, Daniel J Bromberg, Hans Wolff, Linda Montanari, Serheii Vasyliev, Frederick L Altice

**Affiliations:** Department of Medicine, Division of Infectious Disease and Chronic Viral Illness Service, McGill University, Montreal, QC, Canada; Centre for Outcomes Research and Evaluation, Research Institute of the McGill University Health Centre, Montreal, QC, Canada; Department of Social and Behavioral Sciences; University of Heidelberg, Heidelberg, Germany; Yale School of Public Health, Yale University, New Haven, CT, USA; Heidelberg Institute of Global Health, Department of Psychiatry and Psychotherapy (Campus Charité Mitte), Charité—Universitätsmedizin Berlin, Berlin, Germany; Division of Prison Health, Geneva University Hospitals & University of Geneva, Geneva, Switzerland; European Union Drugs Agency, Lisbon, Portugal; Health Care Center of the State Criminal Executive Service of Ukraine, Kyiv, Ukraine; Department of Epidemiology of Microbial Diseases; Department of Medicine, Section of Infectious Diseases, Yale School of Medicine, Yale University, New Haven, CT, USA

## Abstract

The 1990 resolution by the UN General Assembly committed member states to provide health-care equity for people in prison, who are included in the global goals to control HIV and eliminate hepatitis C virus (HCV) by 2030. WHO has set ambitious HCV elimination targets by including people who inject drugs (PWID), yet has not prioritised PWID who are incarcerated, a substantial population who have or are at risk for HCV infection. Human rights principles of health-care equity stipulate that “prisoners should enjoy the same standards of health care that are available in the community, without discrimination on the grounds of their legal status”. Globally, only nine countries provide prison-based needle and syringe programmes (PNSPs), essential evidence-based interventions to holistically reduce the harms from drug use, of which only three countries extend reach to all prisons. Even where available, these services are accessed by few participants. PNSPs are recommended as an essential element of an effective HIV and HCV prevention strategy in prisons, and studies have shown that they are key to achieving HCV elimination in carceral settings. This Viewpoint, based primarily on unpublished data from key country-level stakeholders and expert opinion, highlights our perspective that implementation factors related to PNSP delivery in diverse settings likely contribute to low adoption and use of these services by PWID in prisons compared with in the community. However, successful expansion of these evidence-based interventions will depend on political commitment, national surveillance and monitoring programmes, and state-of-the-art implementation science methods, where inputs from multilevel stakeholders should guide improved implementation. Policy makers are urged to create and support opportunities to scale up PNSPs within countries where they exist and expand them to other countries where they are needed to solidify years of commitment towards the 2030 HCV elimination goals.

## Introduction

Globally, international agencies have called for better control or elimination of bloodborne viruses such as HIV and hepatitis C virus (HCV). However, control efforts require reinvigoration for the estimated 40 million people with HIV and 50 million people with chronic HCV infection, as evidenced by the 1·3 million and 1·0 million new annual infections, respectively.^1^ People who inject drugs (PWID) are estimated to contribute to 10% of new HIV infections and 23–39% of new HCV infections. In 2022, 242 000 preventable deaths from HCV-related cirrhosis and hepatocellular carcinoma occurred.^2^ WHO’s early global HCV elimination targets to treat 80% of people with HCV infection and reduce HCV incidence and liver-related mortality by 90% and 65%, respectively, were revised in 2022 to now reduce incidence to five cases per 100 000 people and mortality to two per 100 000 people by 2030.^3^ However, most countries are not on track to meet these goals by 2030, in large part due to the disproportionate burden of bloodborne viruses among PWID. HIV and HCV infections among PWID are syndemic with incarceration, which occurs commonly in PWID.^4^ PWID are often marginalised from health care and have not been prioritised over other groups in global HCV elimination efforts.^5^ For example, among the 124 countries with national hepatitis plans, 51 prioritised PWID and only 28 (Australia, Benin, Bhutan, Brazil, Burundi, Canada, Colombia, Dominican Republic, Finland, France, Georgia, Germany, Greece, Guinea Bissau, Iceland, India, Iran, Ireland, Kenya, Mexico, Nigeria, Norway, Pakistan, Peru, Rwanda, Switzerland, the United Kingdom, and the United States of America) prioritised people who are incarcerated.^6^ Prioritising people in prison would enhance opportunities to implement better prevention and treatment.

An estimated 11·5 million people are in prison, with over 30 million people cycling through prisons annually,^7^ a number that is increasing, in part due to the criminalisation of drug use,^8^ especially among PWID. Among the 14·8 million PWID worldwide, most inject opioids (82%) and experience incarceration (58%),^9^ both of which increase the risk of HCV infection.^10^ Some PWID continue injecting drugs within prison, whereas other people initiate drug injection during incarceration. HCV transmission risk is especially heightened during injection within prison as numerous people inject with the same unsterile syringe, often due to a scarcity of injection equipment.^11^ Although maintenance on opioid agonist therapies (OATs) with methadone or buprenorphine substantially reduces risk behaviours and transmission of bloodborne viruses such as HIV and HCV, OATs are only available in prisons in 59 countries and are less effective when drug injection involves psychostimulants.^12^ Consequently, international agencies recommend a combination of evidence-based prevention interventions in prisons, such as OATs and needle and syringe programmes (NSPs).^13^ Furthermore, modelling studies support combination treatment and prevention strategies to achieve HCV micro-elimination in carceral settings.^14^

Despite global reductions in HIV and HCV incidence and mortality, incidence among PWID has not substantially declined over time, with approximately 18% of PWID having HIV and 52% having been infected with HCV.^15^ Few data on HIV and HCV incidence among incarcerated PWID exist; however, studies have found that incarceration of PWID perpetuates transmission of HIV and HCV within prison.^16^ Drug injection within prison is common globally, including in parts of Europe (27–65% prevalence of drug injection in European prisons) with documented outbreaks of HIV and HCV in prison.^17^ Beyond transmission of bloodborne viruses within prison, risk taking and transmission increase further following release to the community, as released PWID enter new injection networks.^16^ Taken together, holistic evidence-based interventions are needed in prisons globally to control HIV and eliminate HCV. In this Viewpoint, based on new unpublished data from key country-level stakeholders and expert opinion, we provide insights into important implementation factors that might contribute to decreased adoption and service use of prison-based needle and syringe programmes (PNSPs), which remain a crucial part of evidence-based interventions to reduce harms from injecting drugs in prison.

## Evidence for needle and syringe programmes

NSPs effectively reduce HIV and HCV transmission among PWID in communities and prisons. Not only do they reduce unsterile drug injection, but they also reduce skin and soft tissue infections, increase service engagement—eg, testing and treatment of HIV and HCV—and increase entry into drug treatment programmes.^18^ NSPs are recommended by international agencies (WHO and the UN) as one of 25 essential elements of an effective HIV and HCV prevention strategy in prisons. Unlike NSPs in communities, PNSPs are far more scarce. Despite a global commitment to the fundamental principle of equivalence of health care for people in prison, codified in the Nelson Mandela rules as “prisoners should enjoy the same standards of health care that are available in the community, and should have access to necessary health-care services free of charge without discrimination on the grounds of their legal status”,^19^ highly effective and numerous NSPs in communities have not been equally translated in prisons, where the risk environment is substantially different. Although a 2023 systematic review of 190 countries found that the global coverage of OATs and NSPs for PWID remains low, NSP availability was substantially lower in prisons relative to communities (nine countries *vs* 92 countries), indicating the inequities in accessing prevention services for incarcerated PWID.^20^

## Challenges in translation of NSPs from communities to prisons

Implementation of NSPs in communities has allowed for considerable innovation. NSPs vary in delivery strategy (storefront, mobile outreach, mail delivery, or vending machines), staff involved (professionals or peers), exchange practices (one-for-one or unlimited), and secondary exchange (services where the provision of sterile injecting equipment is not the primary purpose of the service) and are guided mostly by public health mandates. However, in prisons custody often plays an oversized role in whether PNSPs can exist at all and, if they do, how they are delivered. Moreover, despite great efforts to maintain confidentiality, there is little privacy in prisons. In communities, NSPs are provided as harm reduction delivered by outreach workers rather than medicalised by health-care professionals, whereas in prisons, PNSPs are often overseen by health-care workers. Furthermore, for many prisons, health delivery is overseen by the Ministry of Justice, and medical services are demoted in favour of custodial issues, such as safety, security, and maintaining rules (eg, any drug use is illegal) and order (eg, punishment rather than treatment or prevention). Thus, it is likely that PNSPs will require input from multiple stakeholders.

## Variable implementation of PNSPs globally

Table 1 provides important insights from prisons with PNSPs that, when combined with the unique implementation environment in prisons, could assist in future implementation. Despite NSPs being evidence-based prevention interventions for HIV and HCV, PNSPs are available in only nine countries globally, with adoption in only a few countries over the past decade (eg, Ukraine in 2023) despite the increasing incarceration of PWID in many countries. The scale-up of PNSPs remains markedly unchanged and might, in part, be due to large variability in implementation. For example, only one (<1%) of Germany’s 210 prisons provides PNSPs, whereas they are available in all 152 prisons throughout Spain. However, optimal PNSP implementation involves input from multiple stakeholders, including people in prison themselves, medical and custodial staff who oversee them, prison and health administrators, and the society that governs the politics of incarceration. Moreover, implementation strategies might influence delivery of PNSPs. For example, most strategies involving PNSPs involve delivery by prison health-care professionals, yet dispensing machines anonymously dispense paraphernalia in Germany and in some Swiss prisons. In Moldova, PNSP delivery is operated by peers. In Kyrgyzstan, PNSPs are operated by medical professionals, yet informal prison hierarchies (ie, gangs) oppose the programmes and undermine reach to potential clients.^33^ Where offered, PNSPs are available to all PWID without restriction, aside from in Canada, where eligibility is contingent on a complex pre-approval process that delays participation and has restricted access to approximately 75% of applicants due to safety concerns.^34^ Despite variable implementation, use and adoption could be improved through higher quality service delivery; yet data are often scarce and of variable quality to guide process improvement, which, in turn, could improve perception by stakeholders (eg, society and custodial personnel).

For example, data on PNSP reach (the proportion of incarcerated PWID who access PNSPs) or programme-level coverage (number of sterile syringes per person per year) are seldom available, apart from syringe coverage data from Spain for 1992–2009 (table 2). Accurate data are key to better implementation, yet in the absence of data, public health authorities cannot monitor and evaluate the success of PNSPs in reaching those most likely to transmit HIV and HCV during and after incarceration. Even in settings that have provided PNSPs for years, discontinuation or reduction in PNSPs has impacted reach in Germany and Kyrgyzstan, while coverage levels have declined in Spain, potentially due to lower injection levels within prison as OATs have scaled up. However, elsewhere data suggest increasing injection of psychostimulants that would not be affected by OATs.^9,18^ In most countries with PNSPs, these programmes remain as pilots or do not expand to other prisons, reaching few PWID who might benefit from these services within prisons and undermining the sustainability of existing PNSPs. Unlike elsewhere, PNSPs throughout Switzerland continue to expand and Ukraine has newly introduced them.

## Absence of indicators to measure progress towards HIV and HCV elimination among incarcerated PWID

Although WHO has set ambitious targets for the elimination of HIV and viral hepatitis among PWID by 2030,^37^ incarcerated PWID have not been prioritised. International prevention targets suggest that PWID should be able to access 300 needles or syringes per year to reduce new HCV infections to two per year per 100 people by 2030. In the absence of accurate data about injection frequency in prison, which is often reduced compared with the community, there is a need to revise coverage levels to the unique carceral environment.

Table 2 shows key PNSP indicators among the nine countries with PNSPs related to participation, number (and proportion) of needles exchanged and returned, and changes to HIV and HCV prevalence and incidence. Unfortunately, no country has systematically collected data across these key PNSP indicators, and where data are collected, programmes fall well below existing community WHO coverage indicators, suggesting there is ample opportunity to improve access through improved implementation. National surveillance and monitoring systems (eg, PNSP dashboards) are urgently needed to guide better implementation through audit and feedback strategies. National targets should be set for PNSPs, aspirationally at WHO coverage recommendations and minimally to eliminate opportunities for transmission. PNSPs should be optimally designed when authorities accept that people in prison inject drugs and that PNSPs, or even safe consumption sites, are the only ways that this can be done safely. Prioritising this goal, combined with process improvement activities to improve the quality of PNSP delivery, will ensure better scale-up so that more clients can access and remain engaged in harm reduction until they find alternative (eg, OATs) prevention options.

Many factors have undermined adoption, implementation, and sustainability of PNSPs, including political and ideological opposition, legal and regulatory barriers, stigma and cultural resistance, security concerns, resource constraints, lack of awareness and education, restrictive international and national policies, and, importantly, inconsistent implementation and coverage.^38^ PNSPs remain unpopular with multilevel stakeholders, mostly because they are implemented in ways that cause the programme to be marginalised within the prison, rather than advertised as an essential medical service. Consequently, scale-up of PNSPs remains minimal and, in some settings, PNSPs have been abandoned,^39^ with five countries discontinuing them for a myriad of reasons (table 3). The most common reason for PNSP discontinuation was failure to protect participant confidentiality. Had clients been involved in designing or improving the programme, such an outcome might have been avoided. Lack of funding resulted in PNSP discontinuation in Armenia and Romania; however, one cost-effectiveness analysis of PNSPs in Canada provided supportive evidence for both the economic and health benefits of PNSPs related to HCV and injection-related infections.^42^

## Urgent need for better implementation to scale up PNSPs

Given the low levels of adoption, scale-up, and sustainability of PNSPs, now more than ever is it crucial to leverage state-of-the-art implementation science methods, especially given the contextual differences between prisons and the community. At a minimum, improving PNSP reach requires understanding and involving multilevel stakeholders (eg, people in prison and health-care and custodial services), fixing key problems that restrict service delivery, picking a powerful change leader committed to the programme, getting ideas from other settings as implementation differs substantially between sites, and measuring small incremental changes in service delivery to move towards measurable goals. Multilevel stakeholders provide a broad array of perspectives with regard to barriers and potential solutions to a stated expectation (eg, how to better engage more PWID in PNSPs or how to expand PNSPs to other prisons). Although setting expectations is important, there must be support to guide this process, either through coaching or expert facilitation. The Expert Recommendations for Implementing Change (ERIC) project compiled a comprehensive list of implementation strategies that could be used to guide the process,^43^ but that are mostly not used.

These implementation strategies must be tailored to a specific context by understanding and responding to the multilevel barriers and solutions to PNSP service delivery. At an individual level, most research has thus far only focused on incarcerated or released PWID,^44,45^ specifically showing a lack of knowledge and confidentiality concerns, yet the prison risk environment involves meaningful interpersonal relationships with other incarcerated persons (eg, social support and bullying), fear of unsterile syringes,^46^ and mistrust of and stigma and discrimination towards PWID by health-care and custodial staff.^47,48^ Insights into these interpersonal dynamics have resulted in innovative implementation strategies, such as access to vending machines or distribution of needles and syringes through peers. Prison leadership has rarely been the focus of research^49,50^ despite long-standing but mostly unsubstantiated concerns that PNSPs result in increased use of needles as weapons, institutional violence, and needlestick injuries. Few countries with PNSPs report needlestick injuries (table 2), and none reported transmission of HIV or HCV. Custodial staff might not perceive PNSPs as aligned with the mission of prisons and might view them as not promoting recovery and rehabilitation, which could be reframed by supporting PNSPs as a gateway to other services. Health-care providers might perceive provision of sterile equipment as promoting unhealthy behaviours (ie, injecting non-medical substances), yet reframing this as a method to reduce transmission of bloodborne viruses might align this mission. Alternatively, as was done in Moldova, PNSPs can be operated by peers rather than medical staff to reduce bias from health-care professionals. However, reframing might not be sufficient and implementation might require an active process (eg, facilitation) and reconsideration of who delivers PNSP services (eg, nurses, custodial staff, vending machines, or peers), where they are delivered (eg, medical unit or housing unit), how they are accessed (eg, open or restricted to those meeting specific criteria), whether ancillary services are provided (eg, HIV or HCV testing, overdose education and naloxone distribution, OATs, and psychiatric care), and how services are measured (eg, syringes exchanged per person and reductions in unintended harms [overdose, skin and soft tissue infections, and transmission of HIV or HCV]), all of which play a role in organising services.

## Conclusion

Targeting settings where people are incarcerated is central to the global control of HIV and HCV, despite successful scale-up of antiretroviral therapy and direct-acting antivirals in prisons in many settings. However, treatment of bloodborne viruses should be linked to primary and secondary prevention of bloodborne viruses and, ideally, to tailored delivery of PNSPs and OATs in the unique carceral environment. Returning to the basics by using theory-driven and evidence-informed implementation science strategies might be the key to future PNSP adoption, scale-up, and sustainability, but will require a commitment by multilevel stakeholders. By focusing on how we implement and why our strategies work (or do not), we have a higher likelihood of sustainable progress. Until stronger evidence becomes available, policy makers are urged to recognise that injection occurs universally within prisons and not implementing PNSPs has the potential to cause considerable harm, in light of what is known about the risks and benefits of PNSPs and the high prevalence of HIV and HCV among PWID in prisons.

## Figures and Tables

**Table 1:** Landscape assessment of PNSPs in countries with such programmes (updated to Jan 1, 2024) to guide future implementation

	Prevalence of HIV and HCV among people in prison and in the community; prevalence of PWID in the community	Year of PNSP adoption	Total number of prisons in the country; total prison population^7^	Type of prisons with PNSPs; number of male and female prisons with PNSPs	Number of prisons offering PNSPs/total number of prisons	PNSP service delivery model	Any restrictions to participation*; PNSP services available on admission?	Evolution or trajectory of PNSPs
Canada	Prison: HIV 0·92%^21^, HCV 11·0%^22^; community: HIV 5·8%^9^, HCV 45·0%^22^; PWID 0·7%^23^	2018	143 (43 federal; 100 provincial or territorial); 33 833	PNSPs available in federal prisons only; male: 4/37 female: 5/6	Federal: 9/43 (21%); provincial or territorial: 0/100	Prison health-care staff	Restrictions: yes (approval contingent on a required Threat and Risk assessment); available on admission: no	No PNSPs discontinued
Germany	Prison: HIV 0·8–1·3%, HCV 14·3–17·6%^24^; community: HIV 4·1%, HCV 62·9%^9^; PWID 0·24%^9^	1996	210^25^; 40 952	Mixed: 0/39; male: 0/165; female: 1/6 (includes both sentenced and pretrial detainees)	1/210 (<1%)	Dispensing machines (four in total)	Restrictions: no; available on admission: yes	Five PNSPs discontinued in late 1990s due to safety concerns for correctional officers
Kyrgyzstan	Prison: HIV 11·3%, HCV 53·0%^26^; community: HIV 12·4%, HCV 64·7%^9^; PWID 0·68%^9^	2003 (expanded to pretrial detention in 2008)	9; 7633	Male: 8/8; female: 1/1	9/9 (100%)	Prison health-care staff	Restrictions: no; available on admission: yes	Two PNSPs discontinued from pretrial detention facilities in January, 2023, due to reduced levels of incarceration and closure of facilities
Luxembourg	Prison: HIV 2·4%, HCV 13·3–14·5%^27^; community: HIV 1·9%, HCV 81·3%^9^; PWID 0·57%^9^	2005	3; 705	PNSPs available in two prisons and in one pretrial detention centre; male: 1/1, female: 0/0; mixed: 2/2	3/3 (100%)	Prison health-care staff	Restrictions: minimal (written approval by health-care worker, typically a nurse); available on admission: yes	No PNSPs discontinued
Moldova	Prison: HIV not reported, HCV 1·2%^28^; community: HIV 28·3%, HCV 50·0%^9^; PWID 0·4%^9^	1999	17; 6084	PNSPs available in all prisons except prison hospital and juvenile detention centres; male: 10/12, female: 1/1, mixed or pretrial detention centres: 4/4 (100%)	15/17 (88%)	Peer-to-peer anonymous programme delivered through volunteers	Restrictions: no; available on admission: yes	No PNSPs discontinued
Spain	Prison: HIV 4·0%, HCV 2·0%^29^; community: HIV 26·5%, HCV 66·1%^9^; PWID 0·03%^9^	1997 (expanded to all prisons by 2002)	157 (86 in Spain and 71 in Catalonia and Basque Country); 54 197 (excluding Catalonia and Basque Country)	Male: 84/84, female: 7/7, mixed: 66/66	157/157 (100%) [in 2023: 9/86 (10%) distributed needles]; 47/86 (54%) have previously offered PNSPs	Non-governmental organisations	Restrictions: no; available on admission: yes	No PNSPs discontinued
Switzerland	Prison: HIV 0·4%, HCV 6·2%^30^; community: HIV 1·4%, HCV 74·6%^9^; PWID 0·24%^9^	1992	89; 6445	PNSPs available in prisons, including pretrial, sentenced, and semi-open facilities; male: 11/58, female: 1/2, mixed: 3/29	15/89 (17%; 6/26 cantons)	Prison health-care staff and dispensing machines	Restrictions: no; available on admission: yes	No PNSPs discontinued
Tajikistan	Prison: HIV 3·1%, HCV 19·6%^31^; community: HIV 18·0%, HCV 61·3%^9^; PWID 0·46%^9^	2016	19; 14 000	PNSPs available in prisons (n=9) only and not in pretrial detention centres (n=10); male: 3/8, female: 0/1	3/9 (33%)	Prison health-care staff	Restrictions: no; available on admission: yes	No PNSPs discontinued
Ukraine	Prison: HIV 8·.2%, HCV 5·7%^32^; community: HIV 20·4%, HCV 60·6%^9^; PWID 1·01%^9^	2023	85; 43 483	PNSPs available in prisons (n=2); male: 2, female: 0	2/85 (2%)	Prison health-care staff and peer social workers	Restrictions: no; available on admission: yes	No PNSPs discontinued

HCV=hepatitis C virus. PNSP=prison-based needle and syringe programme. PWID=people who inject drugs.

* Anyone who reports past or current injection is eligible.

**Table 2:** Key performance indicators for PNSPs in countries with such programmes (updated to Jan 1, 2024)

	Total number of participants enrolled in PNSPs since inception; average number of participants enrolled in PNSP per month or year	Reach (proportion of incarcerated PWID who access PNSPs)	Coverage (number of needles or syringes distributed per participant per year); total number of needles or syringes distributed per year	Proportion of needles returned in most recent year of PNSP operation	Number of needlestick injuries reported among staff and incarcerated PWID since PNSP introduction to prisons with PNSPs	Number of new HIV or HCV infections since PNSP inception among incarcerated PWID in prisons with PNSPs	Change in HIV or HCV prevalence or incidence since PNSP inception in prisons with PNSPs
Canada	2018–22: 277; NR	NR; 3 of 10 prisons with PNSPs with zero reach since 2018	NR; NR	100%	Staff: 0; PWID: 3 (2017–19)	NR	NR
Germany	NR; NR	NR	NR; 2023: 1483 needles distributed	NR	Staff: 0; PWID: 2	NR	NR
Kyrgyzstan	2016: 1600; 2020: 1150 (decrease over time due to introduction of decarceration programme in 2019); 1441 per year	NR; 2 of 9 prisons with PNSPs with zero reach since 2015	2010–22: 188 needles per participant per year; NR	NR	NR	NR	NR
Luxembourg	2005–22: 383; 22 per year (90% male)	NR	NR; 2005–22: 16 600 needles distributed (average 922 per year); however, important variations between years	>90%	NR	NR	NR
Moldova	Around 75 000; 3000 per year	NR	2021: 18 needles per participant per year; 2021: 2 750 015 needles distributed^35^	90%	Staff: 0; PWID: 0	2018: 30 new new cases; 2022: 25 new HIV cases; HCV NR	From 2007 to 2016 HIV prevalence: 4·2% to 3·8%; HCV RNA prevalence: 21% to 16·2%
Spain	NR; from 1999 to 2009: 20·2 per month	NR	2005: 17·8 needles per participant per year (syringe coverage* 21·6%); 2009: 8·6 needles per participant per year (syringe coverage* 10·4%)^36^; from 1997 to 2021: 219 000 needles distributed; 2022: 948 needles distributed (100% men); 2023: 914 needles distributed (98% men, 2% women)	70·9% (1999–2009)	Staff: 0; PWID: 0	NR	From 1999 to 2009 (Pereiro de Aguiar prison); HIV prevalence: 21% to 8%; HCV RNA prevalence: 40% to 26%
Switzerland	Geneva (2005–22): 383; Geneva (2005–22): 22 per year (90% male)	NR	Geneva: 47 needles per participant per year; Geneva (2023): 2276	Geneva (2023): 99%	Staff: 0; PWID: 0 (in Geneva)	NR	NR
Tajikistan	NR; Oct, 2023: 78	NR	5 needles per participant per week; NR	NR	Staff: 2; PWID: 0	NR	NR
Ukraine	1248; 1070 per year	NR	40 needles per participant per year; NR	80%	NR	NR	NR

HCV=hepatitis C virus. NR=not reported. PNSP=prison-based needle and syringe programme. PWID=people who inject drugs.

* Defined as the number of needles or syringes distributed through PNSP divided by the annual need of needles or syringes in prison.

**Table 3:** Countries where PNSPs have been discontinued

	Year of PNSP adoption	Year of PNSP discontinuation	Number of participants	Reason for PNSP discontinuation
Armenia	2004	2023	NR	Lack of funding from international donors
Iran	2008 (two pilot projects)	2010	NR	Unknown
North Macedonia	2018	2022	NR	Unknown
Portugal	2007 (one pilot project)	2008	0	Confidentiality concerns among participants^40^
Romania	2009	Unknown	0	Lack of funding and confidentiality concerns among participants^41^

NR=not reported. PNSP=prison-based needle and syringe programme.
